# Blueprint for adapting the cardiac rehabilitation model for oncology patients with cardiovascular-kidney-metabolic syndrome

**DOI:** 10.1016/j.ajpc.2026.101658

**Published:** 2026-05-01

**Authors:** Tiffany L. Brazile, Melissa F. Miller, Wendy Johnson, Whittney Trump, Swetha Alluri, Avani D. Rao, Jennifer M. Matro, Ana Barac

**Affiliations:** a Inova Schar Heart and Vascular, Inova Health System, Falls Church, VA, USA; b Inova Schar Cancer Institute, Inova Health System, Fairfax, VA, USA; c Cardiac and Pulmonary Rehabilitation, Inova Health System, Fairfax, VA, USA

**Figure:**
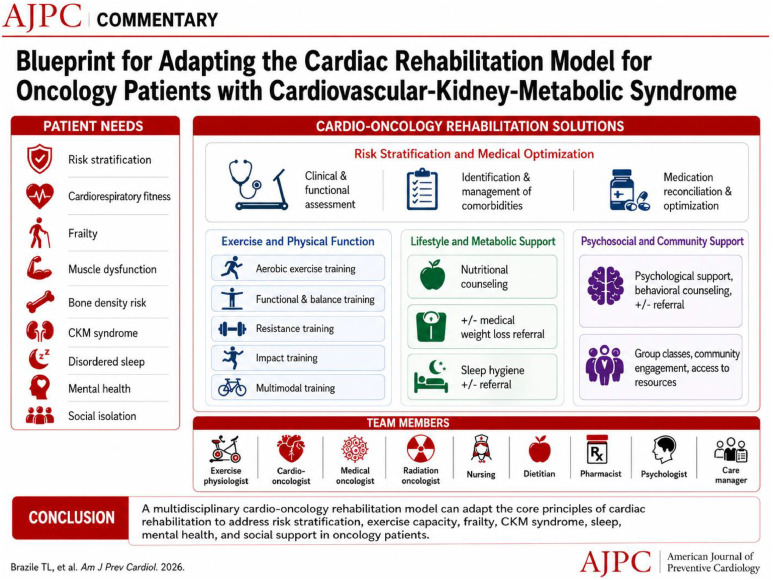
CENTRAL ILLUSTRATION.

## 1 Introduction

Due to advances in early detection, access to care, and cancer treatment, more people are surviving cancers [[1], [2], [3], [4]]. Cancer survivors are at an increased risk of cardiovascular kidney metabolic (CKM) syndrome through shared risk factors (e.g., aging, obesity, insulin resistance, dyslipidemia, physical inactivity, hypertension), treatment-related toxicities, and bi-directional pathophysiological mechanisms (e.g., chronic inflammation, oxidative stress, prothrombotic states, epigenetic dysregulation, clonal hematopoiesis) [[5], [6], [7], [8], [9]]. The rise in cancer survivorship and CKM syndrome represent converging epidemics in which cardiovascular disease remains the leading cause of death [7,8,[10], [11], [12], [13]]. Understanding the increased risk of CKM morbidity and mortality presents an opportunity for this population to benefit from targeted strategies to mitigate disease burden and improve outcomes. It is well known that cardiorespiratory fitness (CRF) is a powerful, modifiable predictor of all-cause mortality, cancer recurrence, and CKM progression [[14], [15], [16], [17], [18], [19], [20]]. Structured exercise is an under-utilized and high-impact intervention with major oncology and cardiovascular benefits [8,[20], [21], [22], [23], [24]]. We summarize the current clinical practice needs and propose the framework for multidisciplinary collaboration in developing integrated, tailored, and multimodal exercise training programs as standard of care.

## 2 Physiologic rationale for exercise in cancer care

Exercise offers a multitude of physiologic benefits for individuals across the cancer continuum through mechanisms that mitigate treatment toxicity, modulate inflammation and immunity, and positively impact CRF, metabolic dysfunction, and psychological outcomes. Randomized controlled trials (RCT) and systematic reviews have consistently demonstrated that exercise during and after cancer treatment is safe and well-tolerated, resulting in improved CRF, strength, fatigue, and anxiety [21,[25], [26], [27], [28]]. Many clinical trials have been conducted in women with breast cancer, with evidence of benefits in patients with other types of cancer (Table 1 [8,24,[29], [30], [31], [32], [33], [34]]). Supervised exercise programs in women with breast and gynecologic cancers show increases in peak oxygen uptake by 2.80 to 3.50 mL/kg/min compared to controls receiving usual care, although results are heterogeneous [22,35]. Notably, compared to cancer-naïve women, anthracycline-treated breast cancer survivors demonstrated an attenuated response to exercise, suggesting treatment-related impairment of exercise adaptations, underscoring the need for tailored exercise programming for this population [36]. The Exercise During Active Surveillance for Prostate Cancer (ERAS) trial, a landmark phase 2 RCT in prostate cancer survivors on active surveillance, found that high-intensity interval training significantly improved CRF and decreased PSA levels compared to usual care [37]. In men with prostate cancer, a systematic review and meta-analysis of randomized controlled trials have shown moderate-to-large improvements in CRF with superior results from aerobic exercise, while resistance training showed increases in lean body mass and leg strength [38]. A comparative study demonstrated that while the effect sizes for response to resistance training was similar between men with prostate cancer on androgen deprivation therapy (ADT) and healthy age-matched controls, the prostate cancer patients only prevented muscle loss while the controls experienced a hypertrophic response [39]. While mechanistically distinct from anthracycline-related attenuations in response to exercise, the impact of ADT further highlights the pressing need for targeted exercise programming for this unique population [40].

**Table 1:** Examples of recent randomized clinical trials that investigated cardiac rehabilitation intervention during and after cancer treatment.

Trial	Study Population (N)	Study design	Duration	CR-intervention	Primary outcome	Secondary Outcomes
**CORE Trial** (Viamonte et al. JAMA Card 2023) [32]	Adult cancer survivors who received cardiotoxic therapy or had preexisting CVD (N=80)	Prospective, single center trial, randomization to center-based CR- intervention vs. community-based exercise training	8 weeks	• Exercise: supervised aerobic + resistance training (60-75min), 2 × /week • Individualized nutritional plan (based on CVRFs) • Psychological management group session (1/week) • Education group session (1/month)	Change in VO_2_peak (by CPET)	Physical function (muscle strength), CVRFs (BP, BMI, lipid profile), body composition, physical activity, QOL, psychosocial distress, intervention adherence, health literacy
**TITAN trial** (Kirkham et al. JACC Adv 2023) [33]	Women with breast cancer (Stage I-III) treated with anthracyclines and/or HER2 targeted therapy (N=80)	Prospective, single center, randomization to multidisciplinary CR-intervention vs. usual care	52 weeks	• Exercise: supervised moderate-intensity aerobic and resistance training (60-90min), 2x/week • Personalized nutritional diagnosis and recommendations (based on patients' assessment and CVRFs)	LVEF (by CMR at 52 weeks)	Cardiotoxicity, changes in GLS, biomarkers (NTproBNP, hsTn, IGF-1), CVRFs (BP, lipid profile, glucose), VO_2_peak, muscle strength, body composition
**ONCORE Trial** (Díaz-Balboa et al. ProgCVDis 2024) [34]	Women with early-stage breast cancer receiving anthracyclines or HER2-targeted agents (N=122)	Prospective, single center, randomization to exercise-based CORe program vs. usual care with PA advice via phone	3-12 months (based on chemotherapy duration)	• Exercise: supervised strength and aerobic training (60min) 2x/week • Dietary advice* • Education and management of CVRFs* *provided to patients in both arms	Cardiotoxicity (defined as ≥10% LVEF decrease to <53% and/or >15% decrease in GLS from baseline) or HF symptoms	Biomarkers (NT-proBNP, Tn), VO_2_peak (by CPET or estimated by 6MWD during COVID19-pandemic), muscle strength, QOL, PA, adherence to recommended diet and exercise

CR: cardiac rehabilitation, CVD: cardiovascular disease; CPET: cardiopulmonary exercise test; CVRFs: cardiovascular risk factors; VO_2_peak: peak oxygen uptake; BP: blood pressure; BMI: body mass index; QOL: quality of life; LVEF: left ventricular ejection fraction; CMR: cardiac magnetic resonance; hsTn: high sensitivity troponin; IGF-1: insulin growth factor-1; CORe: cardio-oncology rehabilitation; PA: physical activity; HF: heart failure; 6MWD: six-minute walk distance.

Combined aerobic and resistance training programs, in concert with dietary interventions, in overweight and obese breast cancer survivors have resulted in significant reductions in visceral adipose tissue, and improvements in glucose regulation, insulin sensitivity, and triglyceride levels [22,[41], [42], [43]]. Responses are dependent on exercise duration, frequency, and volume [44,45]. Potent anti-inflammatory effects have also been observed with aerobic and resistance training in this population, with effects in overweight and obese individuals driven by weight loss [[46], [47], [48], [49]]. The Breast Cancer WEight Loss (BWEL) trial is the largest randomized trial investigating lifestyle intervention in breast cancer patients. While the primary survival analysis is ongoing, the trial showed the feasibility of a remote weight loss intervention combining exercise, diet, and health education [50,51].

Beyond symptom management, observational studies have shown that higher levels of post-diagnosis physical activity are associated with lower risks of cancer recurrence and death [23]. Recently, the well-designed CHALLENGE randomized controlled trial in patients with nonmetastatic colon cancer demonstrated that participation in a three-year supervised aerobic exercise intervention following chemotherapy was associated with a reduced incidence of distant metastatic disease and second primary cancers, as well as a 37% improvement in overall survival [14]. Multiple studies have found that exercise, including resistance training and high intensity interval training, induces direct anti-cancer effects through myokine secretion and enhanced anti-tumor immunity [[52], [53], [54]]. Indeed, these multifaceted mechanisms have been proposed as potential drivers of the observed associations between higher CRF and muscle strength and reduced cancer recurrence, as well as lower cancer-specific and all-cause mortality [17,[55], [56], [57]]. Importantly, exercise is emphasized as a core component of cardiovascular disease prevention in cancer patients and survivors by the multiple scientific societies and organizations, including the National Comprehensive Cancer Network (NCCN), the American Society of Clinical Oncology (ASCO), the American College of Sports Medicine (ACSM), and the American Heart Association (AHA) [8,25,58,59]. Taken together, this growing body of evidence underscores the critical need to integrate exercise into routine oncology care to improve both the quality and quantity of life for individuals with cancer.

## 3 Limitations of current oncology models for exercise as prevention and need for multidisciplinary integration

Despite this strong evidence base, integration of exercise into routine oncology care in the United States remains limited. Most patients with cancer do not have systematic access to evidence-based exercise programming that is appropriately prescribed, monitored, and tailored to their treatment status and symptom burden. Recent guidance emphasizes that exercise interventions must include not only the correct prescription, defined by frequency, intensity, time, and type (FITT), but also behavioral counseling to support adherence and long-term engagement [60]. Delivering such multicomponent programs requires infrastructure that is often absent in community cancer centers, where the majority of patients receive care [8,24,61,62].

A major limitation of current models is the lack of oncology-specific exercise expertise embedded within cancer care teams. Patients undergoing cancer treatment frequently present with complex clinical considerations, including neutropenia, lymphedema risk, bone metastases or osteoporosis, cardiotoxicity, peripheral neuropathy, frailty, and treatment-related fatigue [8]. These factors necessitate specialized exercise assessment and prescription that go beyond general fitness guidelines. In the absence of trained exercise oncology professionals, clinicians may hesitate to recommend exercise, and patients may default to inactivity during a period when physical decline and symptom burden are greatest.

Infrastructure challenges further impede implementation. Evidence-based exercise oncology programs require access to trained personnel, appropriate physical or telehealth space, and equipment to safely deliver aerobic, resistance, balance, flexibility and impact (bone density) training [21,[63], [64], [65], [66], [67]]. Equally critical are systems that enable identification and triage of patients into the right level of exercise support. A recent call to action statement emphasizes the importance of embedding risk stratification and triage questions into clinical workflows—ideally within the electronic medical record (EMR)—to connect patients to appropriate exercise resources based on treatment status, symptom severity, and medical risk [60].

At present, access to exercise services within oncology care is largely restricted to physical or occupational therapy, typically reserved for patients with severe functional impairments or post-treatment complications such as shoulder impairments or lymphedema. These services are often episodic, diagnosis-driven, and associated with insurance barriers and co-pays, limiting their reach [8,62,68]. As a result, many patients who could benefit from proactive, preventive exercise interventions are not served. Ensuring equitable access to exercise oncology programs across the cancer continuum will require new models that extend beyond rehabilitation alone and integrate exercise as a core component of comprehensive cancer care.

## 4 Proposed multidisciplinary cardio-oncology rehabilitation model

Cardiac rehabilitation (CR) is an established, evidence-based model of care that delivers structured exercise training, cardiovascular risk factor modification, nutrition assessment, and psychosocial support for patients with cardiovascular disease [69] (Figure). Despite substantial overlap in symptom burden, functional limitations, and cardiovascular risk profiles, patients with active cancer or cancer survivorship have not traditionally been integrated into CR programs. The siloing of oncology and cardiovascular care has led to a missed opportunity to leverage the existing CR infrastructure to deliver supervised exercise, longitudinal monitoring, and risk reduction strategies to oncology patients. CR’s established safety protocols, exercise facilities, trained personnel, and outcomes tracking systems uniquely position it to support cardio-oncology populations without the need to develop entirely new care models.

The American Heart Association (AHA) Scientific Statement on cardio-oncology rehabilitation outlines a framework for implementing a Cardio-Oncology Rehabilitation (CORE) program within existing CR programs [8]. This approach emphasizes interdisciplinary collaboration, tailored exercise prescriptions, and oncology-specific risk stratification. An integral component of a successful multidisciplinary collaboration includes education of clinical exercise physiologists and cardiac rehabilitation staff on key topics related to cardio-oncology, ranging from infection prevention strategies for immunocompromised patients to management of lymphedema to types of cancer therapies and related cardiotoxicities. Building on the AHA’s proposed model, we emphasize the central role of CORE in addressing complex needs and providing comprehensive cardiovascular and oncology prevention for patients with cancer and survivors (Figure).

Attention to referring the right patient at the right time is integral to ensure that patients who are likely to benefit most from CORE programs receive care during important transitions in care [70]. To accomplish this, targeted referrals to CORE programs for cancer patients with exposure to potentially cardiotoxic chemotherapies or evidence of cardiovascular disease by imaging and/or biomarkers can be integrated into the EMR and occur as part of shared decision making between multidisciplinary oncology providers and patients. Current programs operate within existing, approved CR indications, with the intent to expand eligibility as the growing body of evidence continues to demonstrate improvements in functional capacity and clinical outcomes among diverse oncology populations. While most CR programs are focused on secondary prevention, adaptation of programs for eligible cancer patients offers an opportunity to extend the benefits to primary prevention for those at high-risk (e.g. multiple CKM risk factors) and patients with subclinical cardiovascular disease.

Comprehensive baseline clinical and functional assessments allow CR staff to individualize care. As a patient’s functional limitations and symptoms may fluctuate throughout treatment and recovery, interval evaluations allow for further tailoring of exercise prescriptions to accommodate evolving patient needs. Exercise training can be delivered under established CR safety protocols with attention to immunocompromised status, with individualized progression emphasizing aerobic conditioning, resistance training, balance, and flexibility.

Embedding collaboration between CORE and community-based programs into the care pathway can promote long-term adherence for those needing additional support. Patients can receive education, goal-setting support, and guidance on safe continuation of physical activity and exercise beyond the supervised setting. In addition, shared educational classes offered for oncology and traditional CR participants can extend this reach, create peer support networks, and are resource efficient. These classes address key topics including nutrition, safe exercise at home, cardiac anatomy, cardiovascular risk factor modification, and lifestyle management. Referrals to primary care, preventive cardiology, or other medical subspecialists as indicated can provide additional longitudinal support (Figure).

Implementation of supported, structured exercise programs remains the central challenge in this next era of growing cancer and CKM survivorship. Programs will need to be scalable and, at the same time, individualized based on a patient’s exercise, cancer, treatment, symptoms, and co-morbidity experience. Triage tools are emerging to facilitate connecting the right patient to the right program, recommending a program ranging from oncology rehabilitation, supervised exercise oncology, or community-based programs [60,71] (American College of Sports Medicine: Exercise Oncology Directory. www.movingthroughcancer.org).

## 5 Future directions and call to action

There is a need to establish rigorous evidence of efficacy of a structured exercise program in different cardio-oncology populations and its impact on clinically meaningful cardiovascular endpoints. While several randomized trials have demonstrated that exercise training in cancer patients improves various measures of CRF, components of CKM, and quality of life [22,32,[72], [73], [74]], current evidence is limited by trial size, heterogeneity, and low-to-moderate reporting quality that may limit reproducibility, interpretation, and applicability among different cancer patient populations [22,75,76]. A recent statement emphasizes the importance of inclusion of not only clinical outcomes, but also an assessment of economic outcomes such as the ability to return to work and downstream healthcare expenditures [24]. Demonstrating the value proposition of multidisciplinary CORE programs is a fundamental priority to support reimbursement, sustainability, and future research (Figure).

For cancer patients with CKM syndrome, research should investigate stage-specific exercise interventions alongside other lifestyle interventions such as dietary and behavioral counseling that support both recovery from cancer, related therapies, and comorbid medical conditions that contribute to heightened cardiovascular disease risk [77,78]. Future studies need to examine sex-specific responses, the role of digital health tools to optimize behavior modification, progression of exercise prescriptions, long-term exercise adherence, and patient outcomes.

Implementation research is key to bridging the gap from clinical trials and guidelines to daily practice across the healthcare spectrum [8]. Automating referral systems with the ability to stratify participation data by cancer type, stage, and cardiac risk level would aid in prioritizing access to those who need these programs most. Studies designed to investigate the most effective and efficient delivery practices within multidisciplinary CORE, including remote and hybrid models, are needed to enable care delivery across a variety of settings.

Finally, system-level strategies will be needed to create cost effective partnerships between oncology, cardio-oncology, and cardiac rehabilitation with integration of community programs to maximize patient engagement and ultimately reduce the burden of CKM syndrome in these high-risk patients.

The studies examining exercise prescriptions to improve physical fitness in patients before, during, and after cancer treatment have grown in number and precision as shown in a recent systematic review and meta-analysis of 67 randomized clinical trials (N=4,158 patients) in breast cancer alone [29]. At the same time, research supporting the specific benefits of CR-based interventions in oncology patients is limited. In a systematic search by Fakhraei et al., among 10 studies that utilized CR-based interventions, the reporting quality was low-to-moderate and risk of bias was moderate-to-high, pointing to the limitations of translating their findings into clinical practice [30]. In turn, the International Cardio-Oncology society (ICOS) workgroup on cardio-oncology rehabilitation and exercise (ICOS-CORE) has put forward recommendations for rigorous design of CORE-intervention studies (including tailored exercise prescription, nutritional and psychosocial support, behavioral change, patient education) and standardized outcomes reporting with the goal of facilitating data sharing, comparison, and broad implementation across diverse healthcare settings [24]. Examples of the recent randomized clinical trials that investigated multimodal CR interventions in oncology patients are included in the Table 1. For a comprehensive review of published studies, we direct the reader to meta-analyses and society statements [8,24,[29], [30], [31]].

## Funding

None.

## CRediT authorship contribution statement

**Tiffany L. Brazile:** Writing – review & editing, Writing – original draft, Visualization, Conceptualization. **Melissa F. Miller:** Writing – review & editing, Writing – original draft. **Wendy Johnson:** Writing – review & editing, Writing – original draft. **Whittney Trump:** Writing – review & editing, Writing – original draft. **Swetha Alluri:** Writing – review & editing, Writing – original draft. **Avani D. Rao:** Writing – review & editing, Writing – original draft. **Jennifer M. Matro:** Writing – review & editing, Writing – original draft. **Ana Barac:** Writing – review & editing, Writing – original draft, Conceptualization.

## Declaration of competing interest

The authors declare that they have no known competing financial interests or personal relationships that could have appeared to influence the work reported in this paper.

## Appendix Supplementary materials

Image, application 1
