# Supplementary material for: Blueprint for adapting the cardiac rehabilitation model for oncology patients with cardiovascular-kidney-metabolic syndrome
Source: Am J Prev Cardiol. 2026 May 1;29:101658. doi: 10.1016/j.ajpc.2026.101658 (PMC13329374; doi:10.1016/j.ajpc.2026.101658)
Supplement: Supplementary file 1 [file mmc1.docx]

**Inova Schar Heart and Vascular Institute**

Inova Fairfax Medical Center

8081 Innovation Park Drive

VA 22031

Phone:571-472-2900

Fax:571-472-2901

April 7, 2027

To: Editor in Chief of the American Journal of Preventive Cardiology

Dear Dr Nasir,

We would like to submit a perspective that highlights a major gap in cardiovascular prevention among patients with cancer and cancer survivors. Cardiovascular, kidney, and metabolic risk factors are common among oncology patients and are linked to graded increases in cardiovascular, cancer related, and all-cause morbidity and mortality. The oncology clinical practice guidelines recommend exercise during and after chemotherapy for select cancer types, however, there are limited available resources to guide patients regarding exercise. Cardiometabolic risks remain underrecognized among oncologists and may also be contributing to adverse outcomes.

We propose a framework of utilizing cardiac rehabilitation to address the existing needs and highlight the opportunity for collaboration in addressing cardiometabolic risks and improving overall outcomes in this vulnerable population. Our multidisciplinary team includes experts in cardio-oncology, exercise, cardiac rehabilitation and medical oncology.

We believe that the readership of the American Journal of Preventive Cardiology would find this of interest and we thank you in advance for your consideration and feedback.

Sincerely,

Tiffany Brazile, MD and Ana Barac, MD, PhD

Ana Barac, M.D., Ph.D., F.A.C.C., F.A.H.A.	Tiffany L. Brazile, MD

D’Aniello Chair, Cardio-Oncology	Cardiology Fellow

Inova Schar Cancer	Inova Schar Heart and Vascular

Inova Schar Heart and Vascular	Inova Health System

ana.barac@inova.org	tiffany.brazile@inova.org
